# Currencies of Mutualisms: Sources of Alkaloid Genes in Vertically Transmitted Epichloae

**DOI:** 10.3390/toxins5061064

**Published:** 2013-06-06

**Authors:** Christopher L. Schardl, Carolyn A. Young, Juan Pan, Simona Florea, Johanna E. Takach, Daniel G. Panaccione, Mark L. Farman, Jennifer S. Webb, Jolanta Jaromczyk, Nikki D. Charlton, Padmaja Nagabhyru, Li Chen, Chong Shi, Adrian Leuchtmann

**Affiliations:** 1 Department of Plant Pathology, University of Kentucky, Lexington, KY 40546, USA; E-Mails: juan.pan@uky.edu (J.P.); sflor2@uky.edu (S.F.); farman@uky.edu (M.L.F.); padmaja.nagabhyru@uky.edu (P.N.); li.chen1@uky.edu (L.C.); chong.shi@uky.edu (C.S.); 2 Forage Improvement Division, The Samuel Roberts Noble Foundation, Ardmore, OK 73401, USA; E-Mails: cayoung@noble.org (C.A.Y.); jetakach@noble.org (J.E.T.); ndcharlton@noble.org (N.D.C.); 3 Division of Plant and Soil Sciences, West Virginia University, Morgantown, WV 26506, USA; E-Mail: dan.panaccione@mail.wvu.edu; 4 Advanced Genetic Technologies Center, University of Kentucky, Lexington, KY 40546, USA; E-Mails: jswebb2@uky.edu (J.S.W.); jolanta.jaromczyk@uky.edu (J.J.); 5 School of Pastoral Agriculture Science and Technology, Lanzhou University, Lanzhou 730020, China; 6 School of Grassland & Environmental Science, Xinjiang Agricultural University, Urumqi 830052, China; 7 Institute of Integrative Biology, ETH Zürich, Zürich CH-8092, Switzerland; E-Mail: adrian.leuchtmann@env.ethz.ch

**Keywords:** ergot alkaloids, indole-diterpenes, lolines, Clavicipitaceae, epichloae, endophytes, grasses, *Poaceae*, Poöideae, symbiosis

## Abstract

The epichloae (*Epichloë* and *Neotyphodium* species), a monophyletic group of fungi in the family Clavicipitaceae, are systemic symbionts of cool-season grasses (Poaceae subfamily Poöideae). Most epichloae are vertically transmitted in seeds (endophytes), and most produce alkaloids that attack nervous systems of potential herbivores. These protective metabolites include ergot alkaloids and indole-diterpenes (tremorgens), which are active in vertebrate systems, and lolines and peramine, which are more specific against invertebrates. Several *Epichloë* species have been described which are sexual and capable of horizontal transmission, and most are vertically transmissible also. Asexual epichloae are mainly or exclusively vertically transmitted, and many are interspecific hybrids with genomic contributions from two or three ancestral *Epichloë* species. Here we employ genome-scale analyses to investigate the origins of biosynthesis gene clusters for ergot alkaloids (*EAS*), indole-diterpenes (*IDT*), and lolines (*LOL*) in 12 hybrid species. In each hybrid, the alkaloid-gene and housekeeping-gene relationships were congruent. Interestingly, hybrids frequently had alkaloid clusters that were rare in their sexual ancestors. Also, in those hybrids that had multiple *EAS*, *IDT* or *LOL* clusters, one cluster lacked some genes, usually for late pathway steps. Possible implications of these findings for the alkaloid profiles and endophyte ecology are discussed.

## 1. Introduction

Throughout history, some of the natural products most commonly used and abused by humankind have been the fungus-derived ergot alkaloids (reviewed in [1]). These alkaloids are named after the resting structures (ergots, also called sclerotia), produced by *Claviceps* species on ears of grasses. The ergots are common contaminants of cereals that must be diligently removed to prevent poisoning of food and feed grain supplies. The ergot alkaloids are a diverse group of compounds, and depending on the prominent forms that may occur in contaminated grain, they can cause outbreaks of either convulsive or gangrenous ergotism with devastating results. Ergot alkaloids have also been used for millennia to aid childbirth, and for birth control, treatment of migraines and, recently, treatment of parkinsonism and other CNS disorders. They also are source material for the semisynthetic drug, LSD, by far the most hallucinogenic substance known. In addition to ergot alkaloids, *Claviceps* species can also produce a second class of neurotropic alkaloids, the indole-diterpenes (“tremorgens”), known to cause staggers in livestock [2]. Given the importance and notoriety of *Claviceps* species, it is fitting that they lend their name to the Clavicipitaceae, a large and interesting family of fungi that is surprisingly diverse in host interactions and natural products.

**Figure 1:**
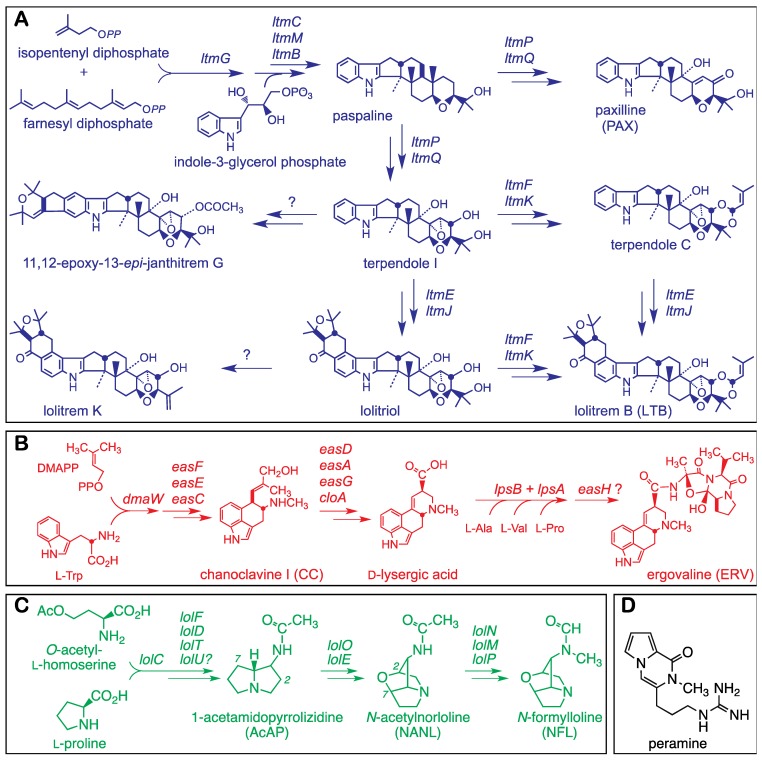
Structures and pathways for alkaloids produced by epichloae (adapted from [6]). Summaries of pathways are shown for indole-diterpenes (**A**), ergot alkaloids (**B**) and lolines (**C**) with structures of major forms of the alkaloids found in grasses symbiotic with epichloae. Panel (**D**) shows the structure of a fourth protective alkaloid, peramine, also produced by many epichloae.

The Clavicipitaceae (Hypocreales, Pezizomycotina, Ascomycota) include pathogens, parasites and symbionts of plants, invertebrate animals, or other fungi, and can produce four different classes of neurotropic alkaloids. Alkaloid structures, and pathways currently deemed likely for three of these classes are shown in Figure 1. The aforementioned ergot alkaloids and indole-diterpenes are produced by many Clavicipitaceae, but are also known from fungi in several other orders of the Pezizomycotina. In contrast, two other alkaloid classes, the lolines and peramine, have a much more restricted distribution and more specific activity against invertebrates. Lolines are potent insecticidal alkaloids [3] that have been found in numerous cool-season grasses (Poaceae, subfamily Poöideae), as well as a spattering of dicotyledonous plants [4]. The reason for their occurrence in the Poöideae has been determined: They are synthesized by symbiotic Clavicipitaceae in the monophyletic “epichloae” (which includes *Epichloë* and *Neotyphodium* species). Similarly, the insect feeding deterrent, peramine, is synthesized by many epichloae, and is known only from cool-season grasses possessing these symbionts. There is ample evidence that many epichloae behave as mutualistic symbionts, and that their capability to make alkaloids that combat herbivores amounts to a major host benefit in mutualism [5].

The epichloae grow systemically in plant aerial tissues by an endobiotic growth habit whereby fungal hyphae invade intercellular spaces and grow as adjacent host cells elongate [7]. Generally there is no obvious external growth from vegetative host tissues, but when host plants produce flowering tillers there is a developmental “decision” as to how the fungus responds. In some cases, every flowering tiller exhibits the epichloid fruiting structure (stroma), which surrounds the immature inflorescence and halts its maturation (choke disease). Ascospores (meiotic spores), and conceivably conidia (mitotic spores) produced on the stromata, can mediate infection of new plants (horizontal transmission) [8,9,10]. In other cases no fruiting structure appears, inflorescences develop normally, and the fungus grows benignly in developing embryos, thereby ensuring transmission from a mother plant to its progeny (vertical transmission). Many epichloae use both transmission strategies, choking some tillers while allowing others to develop normally. However, those epichloae that fail to produce stromata are strictly asexual and—as far as is known—only transmit vertically. The majority of such apparently asexual species described to date have been characterized genetically as interspecific hybrids [11,12]. 

Phylogenetic analysis of housekeeping genes [11,12], along with genome size estimates [13], suggest that such hybrid species have retained most or all of the genome content contributed by two or, sometimes, three ancestral species. Thus, hybridization resulted in diploid or triploid epichloae, whereas the sexual species are haploid. Since there is no evidence that such polyploid hybrids can form easily [14,15], it seems likely that hybridization often leads to increased benefits to the fungus and the host grass, for which there is recent empirical evidence [16,17]. Selosse and Schardl [18] suggest several possible benefits, and one that is relevant to this paper is pyramiding of alkaloid genes for enhanced host protection.

In a survey of epichloae for *in symbio* production of lolines, ergot alkaloids and peramine, most sexual species were found to lack detectable levels, whereas most asexual species produced one or more of these alkaloids [19]. Since that survey, techniques have advanced to detect a broader range of ergot alkaloids, indole-diterpenes and lolines, including intermediates, spur products and end products [4,20,21]. Furthermore, most or all of the genes have been identified for biosynthesis of each of the four alkaloid classes [6]. These advances make it much easier to predict alkaloid profiles on a genetic basis [22,23], and to follow up these predictions with comprehensive chemotyping. 

Surveys of epichloae for the gene clusters encoding biosynthetic enzymes for lolines, (*LOL*), ergot alkaloids (*EAS*), and indole-diterpenes (*IDT*) have led to interesting findings. Most epichloae, both sexual and asexual, have genes for one or more of these classes of protective alkaloids [24]. Furthermore, there is considerable chemotypic variation among isolates of several sexual epichloae, though many isolates lack key genes required to make any of these three kinds of alkaloid. In contrast, most asexual epichloae surveyed to date have genes for one or more of these alkaloids, although there is also chemotypic diversity within some hybrid species. There have been fewer surveys for *perA*, which is required for peramine production, but the limited results so far suggest that peramine is very common, though not universal, among isolates of both sexual and asexual epichloae.

In this study we survey and sequence selected *LOL*, *EAS* and *IDT* genes from representatives of most known nonhybrid species of epichloae, both sexual and asexual, and from several asexual hybrids, in order to identify the sources of these genes in the hybrids. We find evidence that strict vertical transmission may often serve as a selective filter for strains that produce protective alkaloids.

**Table 1:** Species of *Epichloë* and *Neotyphodium* in this study, and their most abundant alkaloids.

				EA ^a^		IDT ^a^		LOL ^a^	
Species and variety or genotype	Isolate ^b^	Reference or PI	Host ^c^	Pre-dicted	Observed	Pre-dicted	Ob-served	Pre-dicted	Observed
*Epichloë amarillans*	E57 = ATCC 200744	[6]	*Agrostis hyemalis*	–	–	–	nt	NANL	NANL
*E. amarillans*	E4668	This study	*Agrostis hyemalis*	ERV	–	–	nt	–	nt
*E. baconii*	E1031 = ATCC 200745	[25]	*Calamagrostis villosa*	–	nt	–	nt	–	nt
*E. brachyelytri*	E4804	[6]	*Brachyelytrum erectum*	CC	nt	–	nt	AcAP	AcAP
*E. bromicola*	E502 = ATCC 200750	[6]	*Bromus erectus*	–	nt	–	nt	–	–
*E. canadensis*	e4815		*Elymus canadensis*	ERV	ERV CC	–	nt	NANL	NANL
*E. canadensis*	CWR5	[26]	*Elymus canadensis*	ERV	ERV CC	–	nt	NANL	NANL
*E. canadensis*	CWR34	[26]	*Elymus canadensis*	CC	CC	–	nt	AcAP	AcAP
*E. elymi*	E56 = ATCC 201551	[6]	*Elymus virginicus*	CC	CC	–	nt	–	–
*E. festucae*	E2368	[6]	*Festuca rubra* and *Lolium giganteum* (ascospore isolate)	ERV	–	–	nt	NFL	NFL NML NAL NANL
*E. festucae*	Fl1	[6]	*Festuca trachyphylla*	ERV	ERV CC	LTB	LTB	–	–
*E. glyceriae*	E277	[6]	*Glyceria striata*	ERV	–	–	nt	AcAP	AcAP
*E. “mollis”*	E3601 = AL9924	This study	*Holcus mollis*	ERV	nt	–	nt	–	nt
*E. poae*	E4646	This study	*Poa nemoralis*	–	nt	–	nt	–	nt
*E. poae*	E5819	[6]	*Poa nemoralis*	ERV	nt	–	nt	–	nt
*E. typhina*	E8 = ATCC 200736	[6]	*Lolium perenne*	–	nt	–	nt	–	–
FaTG-2 G2	NFe45079	[23]	*Lolium* sp. (6x)	ERV	ERV CC	LTB	LTB TD	–	nt
FaTG-2 G3	NFe45115	[23]	*Lolium* sp. (6x)	ERV	ERV CC	TD	TD	–	nt
FaTG-3	NFe1100	This study	*Lolium* sp. (6x)	–	–	TD	nt	NFL	NFL
FaTG-3	e4074	[11]	*Lolium* sp. (6x)	–	–	–	nt	NFL	NFL NML NAL
FaTG-4	e4305	PI 598863	*Lolium* sp. (10x)	ERV	nt	TD	nt	–	–
*Neotyphodium aotearoae*	e899 = MYA-1229	[27]	*Echinopogon ovatus*	–	nt	TD	nt	NFL	NFL
*N. chisosum*	e3609 = ATCC 64037	[28]	*Achnatherum eminens*	–	nt	–	nt	NFL	nt
*N. coenophialum*	e19 = ATCC 90664	[11]	*Lolium arundinaceum*	ERV	ERV CC	–	nt	NFL	NFL NML NAL NANL
*N. coenophialum*	e4163	PI 422777	*Lolium* sp. (4x)	ERV	ERV	TD	nt	NFL	NFL NML NAL NANL
*N. coenophialum*	e4309	PI 598903	*Lolium arundinaceum*	–	–	TD	nt	NFL	NANL
*N. funkii*	e4096	[28]	*Achnatherum robustum*	CC	CC	TD	nt	–	–
*N. gansuense* var. *inebrians*	e818 = MYA-1228	[6,28]	*Achnatherum inebrians*	EN LAH	EN LAA LAH	–	nt	–	–
*N. gansuense*	e7080	[6]	*Achnatherum inebrians*	–	nt	PAX	PAX	–	nt
*N. occultans*	non-culturable	[29]	*Lolium* spp. (2x)	–	nt	PAX	nt	NFL	NFL NML NAL NANL
*N. siegelii*	e915 = ATCC 74483	[11,30]	*Lolium pratense*	–	–	PAX	nt	NFL	NFL NML NAL NANL
*N. uncinatum*	e167 = CBS 102646	[6]	*Lolium pratense*	–	–	–	nt	NFL	NFL NML NAL NANL
*N. lolii* × *E. typhina*	Lp1 = e144	[11]	*Lolium perenne*	ERV	ERV CC	TD	TD	–	–
PauTG-2	e55	[11]	*Poa autumnalis*	nt	nt	nt	nt	NFL	NFL NML NANL

^a^ Abbreviations: AcAP = 1-acetamidopyrrolizidine, CC = chanoclavine, EN = ergonovine, ERV = ergovaline, LAA = lysergic acid amide, LAH = lysergic acid α-hydroxyethylamide, LTB = lolitrem B, NANL = *N*-acetylnorloline, NFL = *N*-formylloline, nt = not tested, PAX = paxilline, TD = terpendoles. - = none detected; ^b^ Strains listed with ATCC and MYA prefixes are in the American Type Culture Collection. CBS = Centraalbureau voor Schimmelcultures, Utrecht, The Netherlands; ^c^ Ploidies of *Lolium* species are indicated as 2x = diploid, 4x = tetraploids, 6x = hexaploid, 10x = decaploid.

## 2. Results

### 2.1. Genome Assemblies and Identification of Alkaloid Genes

**Figure 2:**
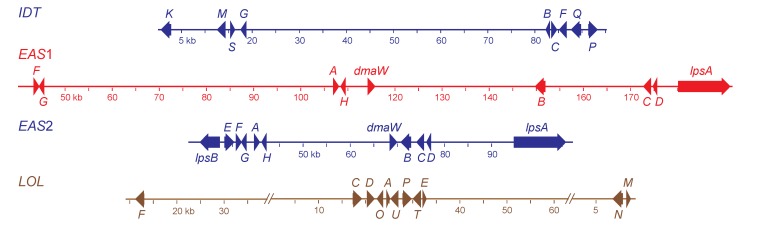
Structures of the alkaloid biosynthesis gene clusters in *Neotyphodium coenophialum* e4163. Maps are color coded according to ancestral clade of origin (see Figure 3). The indole-diterpene (*IDT*) gene cluster was from clade II (*E. festucae*). Each gene in the *IDT* cluster (accession number KC970578) is designated by its final letter, where *K* = *idtK*, *etc.* Two ergot-alkaloid gene clusters were identified, one from clade V (*EAS*1, accession number KC989569), and the other from clade II (*EAS*2, accession number KC989570). Genes in the *EAS* clusters are abbreviated as follows: *A = easA*, *B = cloA*, *C = easC*, *D = easD*, *E = easE*, *F = easF*, *G = easG*, *H = easH*. Other *EAS* cluster gene names are given in full. The loline alkaloid (*LOL*, accession numbers KC990458, KC990457 and KC990459) gene cluster was from clade Ib (*E. poae*). Each gene in the *LOL* cluster is designated by its final letter, where *F* = *lolF*, *etc.*

Isolates of *Epichloë* and *Neotyphodium* species are listed in Table 1, and statistics for genome assemblies are given in Table 2. For genomes with comparable fold-coverage and comparable average read length, the quality of the assemblies depended mainly on the genome size and ploidy. Considering its polyploid nature and large genome size, an exceptionally good assembly was obtained for the *N. coenophialum* e4163 genome, in which three of the four alkaloid gene clusters were contained within individual contigs of greater than 150 kb (Figure 2). One of these contigs, which contained the *IDT* cluster, had a telomeric repeat beginning 565 bp downstream of the *idtK* stop codon. Both the *EAS*1 and *EAS*2 clusters were assembled on separate contigs, and the very close relationship of all *EAS*2 genes with the *E. festucae EAS* genes indicated that the clusters remained intact according to their ancestral relationships. Although the *N. coenophialum* e4163 assembly showed no indication of telomeres linked to the *EAS* clusters, in the assembly of *N. coenophialum* strain e19, both *lpsB*1 and *lpsB*2 were on contigs with telomere repeats at the ends. In both cases, the telomeres were downstream of the *lpsB* gene. The *N. coenophialumLOL* clusters were not completely assembled, but were arranged in three contigs of the e4163 assembly. One contig contained *lolF* and flanking (downstream) housekeeping genes, another contig contained eight genes, *lolC-lolE*, arranged similarly to those in *N. uncinatum* [31], and the third contig contained *lolN* and *lolM*. The arrangement of the *LOL* genes and housekeeping genes flanking *lolF* was consistent with their arrangement in the genome of *E. festucae* E2368 [6].

**Table 2:** Assembly statistics for genomes of *Epichloë* and *Neotyphodium* species.

Strain	Genome assembly size (Mb)	Endophyte ploidy	Fold-coverage	Number of contigs	Contig N50 (bp) ^a^
E8	41.3	1	21.2	2253	42,567
e19	95.4	3	34.4	8808	25,663
E56	31.8	1	25.6	5206	32,484
E57	38.0	1	27.7	1871	49,298
Lp1	51.1	2	7.6	27,822	2228
E277	49.3	1	27.7	2658	42,649
e818	29.8	1	87.3	2519	39,993
Fl1	34.9	1	52.3	1277	84,986
e899	34.3	1	26.3	1848	56,460
E1031	38.0	1	18.5	3358	34,415
E2368	34.7	1	27.8	1316	87,544
E3601	36.1	1	28.0	890	97,803
e3609	108.7	3	10.2	32,104	4981
e4096	61.2	2	29.5	6100	20,015
e4163	97.7	3	32.5	4349	46,910
e4305	76.6	2	21.9	17,406	7250
e4309	81.8	3	14.6	34,254	3494
E4668	40.7	1	25.0	1851	45,395
E4804	44.1	1	24.6	4542	20,998
e4815	63.3	2	13.7	21,592	4186
E5819	34.0	1	28.6	2072	36,475
E7080	39.5	1	41.5	1307	56,237

^a^ N50 is defined as the minimum length of the largest contigs or scaffolds (as specified) that together contain 50% of the genome assembly.

For other genomes, most alkaloid genes assembled well, although whole clusters were not generally assembled on single contigs. In cases where two copies of an alkaloid gene were present, the copies often failed to assemble individually over their whole length. Instead, regions with very high similarity tended to collapse into single short contigs. Nevertheless, inspection of the reads revealed polymorphisms indicative of the two gene copies. These situations arose in polyploid (hybrid) genomes with relatively low fold-coverage, affecting the assemblies of *EAS* genes in *E. canadensis* e4815 and *LOL* genes in *N. chisosum* e3609. To deconvolute the multiple gene copies we inspected the aligned reads in the .ace files generated by the Newbler assembler. For those strains lacking comprehensive genome sequence data, fragments of *tubB* and alkaloid genes were PCR-amplified and sequenced from template DNA derived from cultures or symbiotic plant tissue.

### 2.2. Endophyte Species Relationships and Hybrid Origins of Asexual Epichloae

**Figure 3:**
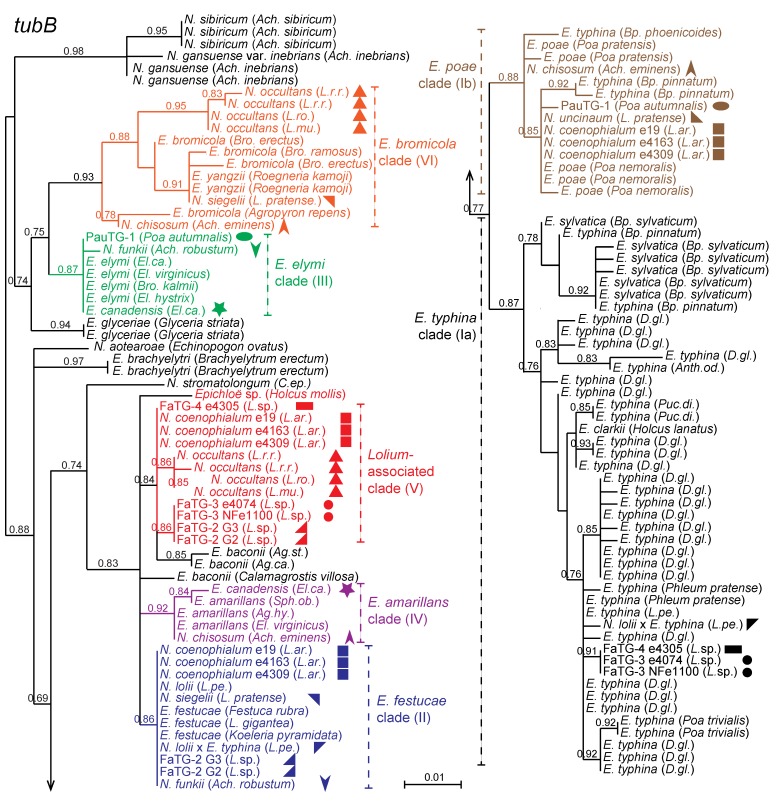
Phylogeny of *tubB* genes from all known haploid (non-hybrid) and ten hybrid epichloae. Where hybrids have multiple gene copies from different ancestral species, the different copies are indicated by symbols of the same shape with different colors. Phylogenetic clades contributing to hybrids are color coded and numbered Ia, Ib, II, III, IV, V, and VI. Supplementary Table S1 contains the *tubB* accession numbers.

Figure 3 presents a phylogeny based on the first three introns of the beta-tubulin gene, *tubB*, relating representatives of sexual *Epichloë* and other nonhybrids to the hybrid species in this study. Clades that include contributors to hybrid genomes are numbered I through VI, in accordance with the mating population designations used prior to formal naming of the sexual species [32]. In some cases these clades correspond to individual sexual species, such as *E. festucae* (clade II), *E. elymi* (clade III) and *E. amarillans* (clade IV). In contrast, clade I has an especially wide diversity, includes isolates from a broad range of grasses within the Poöideae, and encompasses four named *Epichloë* species. Members of three of the clade I species, *E. typhina*, *E. clarkii* and *E. poae*, are interfertile, whereas the fourth, *E. sylvatica*, apparently is not interfertile with *E. typhina* [25]. Because of their importance in the origins of hybrid species, a major clade of *E. typhina* has been designated as clade Ia, and those in the newly describe *E. poae* are in clade Ib [10]. The *E. bromicola* clade (clade VI) includes *E. yangzii* [33], which may be considered a synonym of *E. bromicola* [34,35]. 

Hybrid endophytes tend to have multiple copies of housekeeping genes [11]. With the exception of *N. uncinatum*, all of the hybrid endophytes in this study had two or three *tubB* genes, sequences of which were in distinct clades (Figure 3). In past studies, the phylogenies of *tubB* and genes for translation elongation factor 1-α (*tefA*) and γ-actin (*actG*) supported the same hybrid origins for each of these species [11,28]. In general, the hybrids are asexual, although some asexual epichloae appear to be haploid and not hybrid in origin. 

Most of the broad-leaf members of *Festuca/Lolium/Schedonorus* grass complex, such as tall fescue, meadow fescue and perennial ryegrass, have asexual endophytes, which in turn are restricted to individual grass species (see Table 1). One species, *Neotyphodium lolii*, is an asexual derivative of *E. festucae* (clade II) that is by far the most common symbiont of *Lolium perenne* (perennial ryegrass). This grass also can host *Epichloë typhina*, as well as an asexual *N. lolii* (clade II) × *E. typhina* (clade Ia) hybrid (exemplified by isolate Lp1). Perennial ryegrass is closely related to other diploid species including *Lolium pratense* (=*Schedonorus pratensis*; meadow fescue) and the various species of annual ryegrass. The most common endophyte of meadow fescue is *N. uncinatum*, a hybrid of *E. bromicola* (clade VI) and *E. poae* [10]. A second endophyte from meadow fescue is *N. siegelii*, a hybrid of *E. bromicola* (clade VI) and *E. festucae* (clade II). *Neotyphodium occultans* is the most common endophyte in the annual ryegrass species, *Lolium multiflorum*, *L. temulentum*, *L. canariense*, and *L. rigidum.* This endophyte has an *E. bromicola* (clade VI) genome along with a second genome from the “*Lolium*-associated endophyte” clade that is most closely related to *E. baconii* isolates from *Agrostis* species (clade V). Genomes from clade V appear in four other hybrid epichloae, all endophytes of grasses in the *Lolium arundinaceum* (=*Schedonorus arundinaceus*; tall fescue) species complex. The most complex hybrid, *N. coenophialum*, has genomes from clade V, *E. festucae* (clade II) and *E. poae* (clade Ib), and has been found in hexaploid (6x) tall fescue from northern Europe and from the Atlas Mountains of North Africa [36]. Two undescribed hybrid species, designated FaTG-2 and FaTG-3, have been isolated from Mediterranean 6x tall fescue, and two chemotype variants, G2 and G3, have been described previously [23]. Another hybrid species that we designate FaTG-4 (referred to as FaTG-3-like in Ekanayake *et al*. [37]) was isolated from a decaploid (10x) tall fescue from Morocco. FaTG-2 is a clade V × clade II hybrid. FaTG-3 and FaTG-4 are clade V × clade Ia hybrids, distinguishable by variations in their clade V gene sequences and alkaloid profiles.

Genome sequences were also obtained from two endophytes of *Achnatherum* species (tribe Stipeae). The *tubB* and other phylogenies (Figure 3, Table 3) indicated that *N. chisosum* from *Ach. eminens* and *N. funkii* from *Ach. robustum* had different origins [28]. Interestingly, each of these endophyte species was a hybrid with ancestors related both to North American species (*E. elymi* clade III, *E. amarillans* clade IV) and Eurasian species (*E. bromicola* clade VI, *E. poae* clade Ib, and *E. festucae* II). These results suggest that the ancestral species previously had overlapping geographical ranges.

**Table 3:** Origins of housekeeping and alkaloid gene loci in hybrids ^a^.

Species	Isolate	*tefA*	*tubB*	*EAS*	*IDT*	*LOL*
*E. canadensis*	e4815	III,IV	III,IV	III,IV	–	IV
*E. canadensis*	CWR34	III,IV	III,IV	III	–	IV
*N. lolii* ×* E. typhina*	Lp1	Ia,II	Ia,II	II	II	–
FaTG-2 G2	NFe45079	II,V	II,V	II,V	II,V	–
FaTG-2 G3	NFe45115	II,V	II,V	V	V	–
FaTG-3	NFe1100	Ia,V	Ia,V	–	V	Ia
FaTG-4	e4305	Ia,V	Ia,V	V	V	–
*N. chisosum*	e3609	Ib,IV,VI	Ib,IV,VI	–	–	IV,VI
*N. coenophialum*	e19	Ib,II,V	Ib,II,V	II,V	(II)	Ib
*N. coenophialum*	e4163	Ib,II,V	Ib,II,V	II,V	II	Ib
*N. coenophialum*	e4309	Ib,II,V	Ib,II,V	–	II	Ib
*N. funkii*	e4096	II,III	II,III	III	II	–
*N. occultans*	various	V,VI	V,VI	–	V,(VI)	VI
*N. siegelii*	e915	II,VI	II,VI	–	II,(VI)	VI
*N. uncinatum*	e167	Ib	VI	–	–	Ib,VI
PauTG-1	e55	Ib,III	Ib,III	–	–	Ib

^a^ Clades are: *E. typhina* clade Ia, *E. poae* clade Ib, *E. festucae* clade II, *E. elymi* clade III, *E. amarillans* clade IV, *E. baconii/Lolium*-associated clade V, *E. bromicola* clade VI. Clades represented in parentheses indicate complete cluster was not found from that clade.

Phylogenetic analysis of genes from *E. canadensis*, an endophyte of the host grass *Elymus canadensis* (tribe Triticeae), supported hybrid origin from two North American ancestors, *E. elymi* (clade III) and *E. amarillans* (clade IV) (Figure 3) [26]. Both of these ancestral species have been isolated from the related host, *Elymus virginicus* [11], although only *E. elymi* has so far been observed to fruit on *Elymus* species. 

### 2.3. IDT Gene Profiles and Origins in Hybrid Epichloae

**Figure 4:**
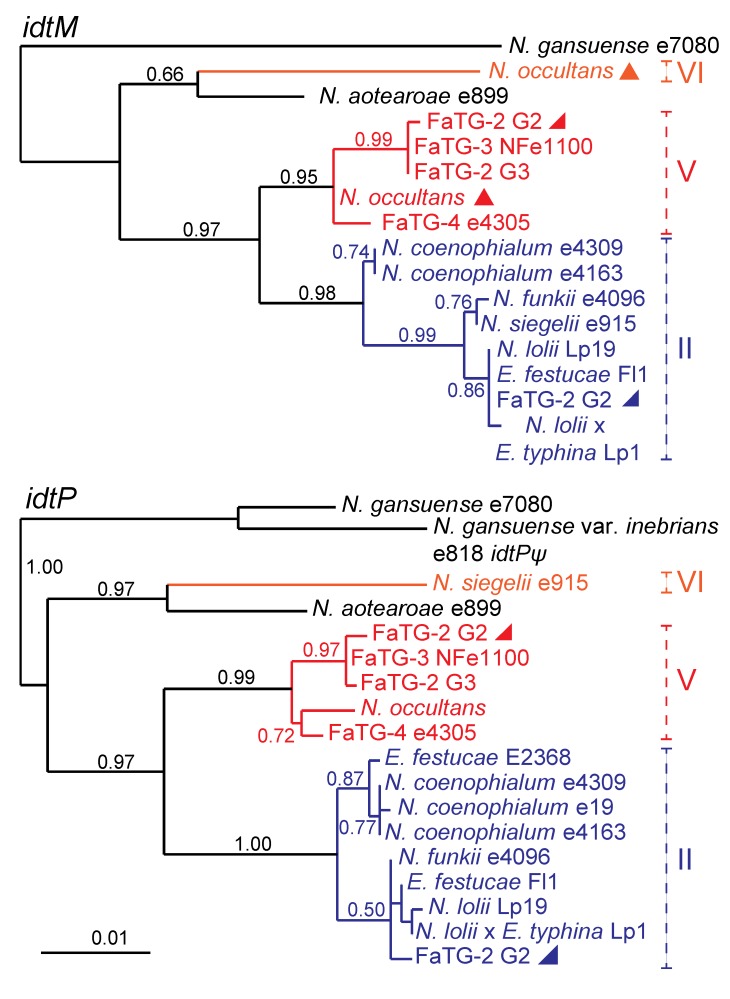
Phylogeny of two *IDT* genes, *idtM* and *idtP* in epichloae. Inactive genes (pseudogenes) are labeled ψ. Clades and multiple gene copies are labeled as in Figure 3. Supplementary Table S2 contains the *IDT* accession numbers.

Origins of the *IDT* genes in the hybrid species were evident from phylogenetic analysis. The *idtM* and *idtP* phylogenies are provided in Figure 4, as examples. The *E. festucae* ancestors of *N. coenophialum* strains e4309 and e4163, *N. funkii* e4096, and *N. lolii* × *E. typhina* Lp1, provided each of these hybrids with a complete complement of *IDT* genes except for the lolitrem B-specifying genes, *ltmE* and *ltmJ*. Therefore, these hybrids may produce terpendoles or other indole-diterpenes, and apart from Lp1 [22] their chemotype profiles have yet to be determined. A third *N. coenophialum* strain, e19, had only *idtP*, which also was apparently derived from the *E. festucae* ancestor. None of these three hybrid species had *IDT* genes from ancestors other than *E. festucae*. 

In contrast, *N. siegelii* appeared to have acquired most *IDT* genes (at least *idtG*, *idtC*, *idtB*, *idtS*, *idtM*, *idtF*, and *idtK*) from its *E. festucae* ancestor, and *idtP* from another source, possibly but not definitively identified as itsclade VI ancestor (Figure 4). Also in the *N. siegelii idtF* gene a single base deletion was identified that should cause a frame shift and early termination, rendering the encoded protein nonfunctional. An identical mutation has been identified in *E. festucae* isolates Fg1 (host *Festuca glauca*) and Frc7 (host *Festuca rubra* subsp. *commutata*), which produce mainly lolitrem K and the pathway intermediate, paxilline, respectively [22]. 

*Neotyphodium occultans* and three other distinct hybrid species, tentatively designated FaTG-2 (variants G2 and G3), FaTG-3, and FaTG-4, seemed likely to derive their *IDT* genes from the clade Vancestor. In addition, *N. occultans* had two copies of *idtC* and *idtM*, where the second copy seemed likely to be derived from *E. bromicola* (clade VI). Also, no *idtK* gene was identified in *N. occultans*. However, comprehensive analysis of alkaloid clusters from *N. occultans* was difficult because this species is not culturable, and we could not rule out the possibility that *idtK* and clade VI copies of other *IDT* genes failed to yield PCR products due to differential primer specificity. FaTG-2 genotype G2 isolates, which produce lolitrems [23], also had *IDT* gene copies derived from clades II and V, and their *ltmE* and *ltmJ* genes were from clade II (*E. festucae*). 

Results of phylogenetic analysis of all *IDT* genes for all isolates with sequenced genomes (listed in Table 2) were consistent with their hybrid ancestors, with the sole exception of *N. aotearoae* e899, an asexual but nonhybrid epichloid endophyte. Phylogenetic relationships of five *IDT* orthologs, *idtG*, *idtC*, *idtM*, *idtS*, and *idtP*, and two pseudogenes, *idtB* and *idtF*, were consistent with the relationships of *N. aotearoae* housekeeping genes such as *tubB* (see, for example, Figure 4). Four other genes, which we tentatively designated *idtB*-like, *idtF*-like, *idtK*-like and *idtQ*-like, were embedded in AT-rich repetitive sequences, and none assembled on the same contigs as the five *IDT* gene orthologs. These four *idt*-likegenes in *N. aotearoae* were highly divergent from their closest homologs in other epichloae, suggesting that they may be xenologs (homologs resulting from horizontal gene transfer) or ancient paralogs. 

### 2.4. EAS Gene Profiles and Origins in Hybrid Epichloae

**Figure 5:**
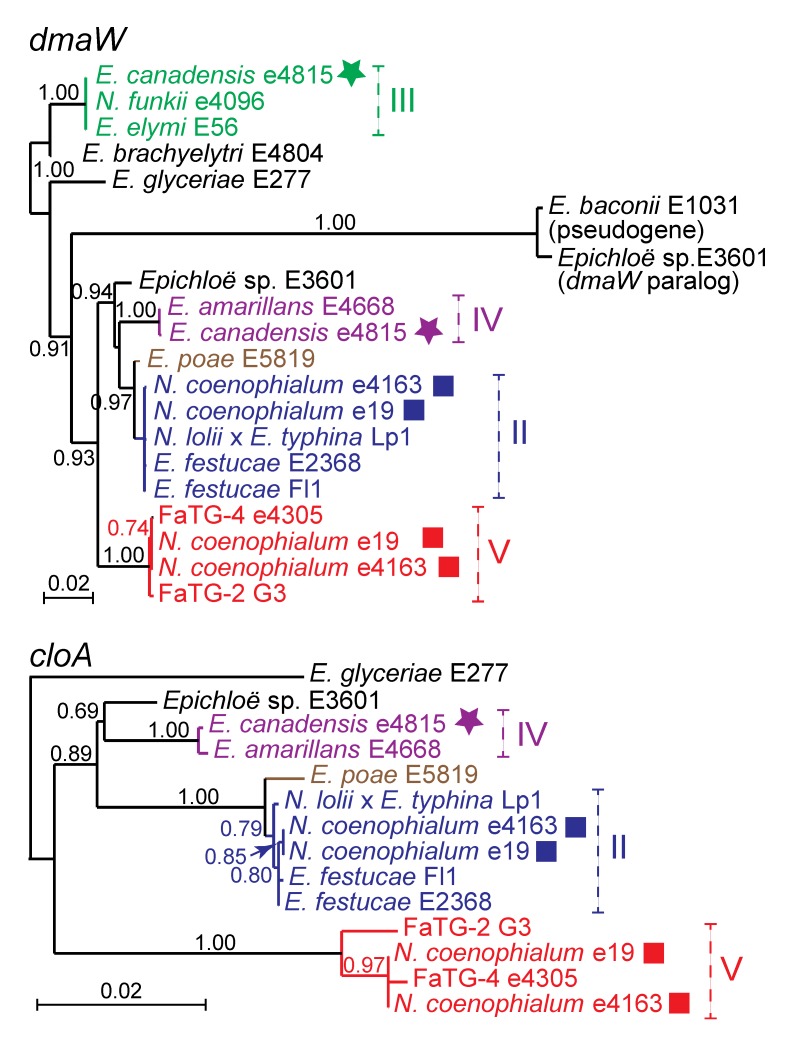
Phylogeny of two *EAS* genes, *dmaW* and *cloA*. Clades and multiple gene copies are labeled as in Figure 3. Supplementary Table S2 contains the *EAS* accession numbers.

The *EAS* genes of hybrid epichloae, like the *IDT* and *LOL* genes, were derived from ancestors identifiable by comparing the *EAS* and housekeeping gene phylogenies. Comprehensive phylogenetic analysis of all *EAS* genes for all isolates with sequenced genomes (see Table 2) indicated relationships consistent with their hybrid origins. As an example, *dmaW* and *cloA* phylogenies are given in Figure 5. (For this analysis, the genes from *N. gansuense* var. *inebrians* E818 were omitted because its *EAS* cluster appears from phylogenetic and structural analysis to be much more similar to those of *Periglandula* and *Balansia* species than to those so far identified in all other epichloae.) Interestingly, paralogous *dmaW* genes were identified in two sexual strains, *Epichloë* sp. E3601 (from the host grass *Holcus mollis*) and *E. baconii* E1031 (from *Calamagrostis villosa*). These paralogs were not located in *EAS* clusters, and though closely related to each other, they were very divergent from *EAS*-cluster *dmaW* genes. Nevertheless, they grouped within the clade of *dmaW* genes from epichloae (data not shown), and for that reason appeared to have arisen from the genes encoding authentic dimethylallyltryptophan synthase. Another surprising result was the close relationship of *E. poaeEAS* genes to those of *E. festucae*, in stark contrast to the housekeeping gene relationships.

Clades II, III, IV and V all contributed *EAS* clusters to hybrids (Figure 5). Half of the 12 hybrid species investigated had *EAS* clusters, and each of three hybrid species, *E. canadensis*, *N. coenophialum* and FaTG-2 (G2), had *EAS* clusters from two different ancestors. Interestingly, *E. canadensis* e4815 contained a complete *EAS* cluster from the *E. amarillans* ancestor but only four *EAS* genes (*dmaW*, *easE*, *easF* and *easC*) from the *E. elymi* ancestor, while the other *E. canadensis* isolate CWR34 only contained the same four *E. elymi EAS* genes (Table 3). This was in keeping with the composition of *EAS* clusters identified in isolates of *E. elymi* (E56) and *E. amarillans* (E4668). 

### 2.5. LOL Gene Profiles and Origins in Hybrid Epichloae

**Figure 6:**
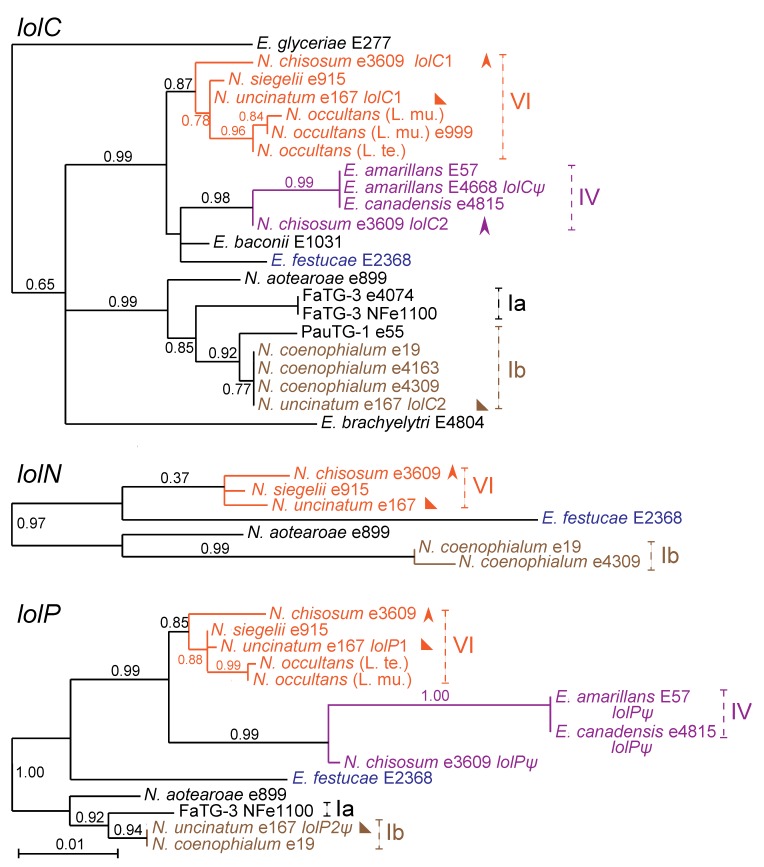
Phylogeny of three *LOL* genes, *lolC*, *lolN*, and *lolP*. Pseudogenes of *lolC* and *lolP* are labeled *lolC*ψ and *lolP*ψ, respectively. Clades and multiple gene copies are labeled as in Figure 3. The *lolN* gene of FaTG-3 was not sequenced. Supplementary Table S2 contains the *LOL* accession numbers.

The relationships of loline biosynthesis genes in hybrids were entirely consistent with the ancestral genome contributions as discerned from the *tubB* phylogeny. This was true for *lolC*, *lolN*, and *lolP* (Figure 6) for all loline producers listed in Table 3, and for all *LOL* genes for all isolates with sequenced genomes (see Table 2). 

Even though no *LOL* genes were identified in two ancestral species, *E. bromicola* and *E. poae*, common relationships of *LOL* genes in several hybrids indicated their origin from these species or close relatives (Figure 6). The number of hybrid species with *LOL* clusters from *E. bromicola* (four) or *E. poae* (three) was surprising given the apparent rarity of *LOL* in the corresponding sexual species. In a survey of 10 plants of three species with *E. bromicola* endophytes, no loline alkaloid producer was identified [19], and in a survey of 50 *Poa nemoralis* plants with *E. poae*, we found no *LOL* genes. Nevertheless, contributions of *E. bromicola* and *E. poae* ancestors could be inferred from the *LOL* gene phylogenies (Figure 6). *LOL* clusters in four hybrids, *N. chisosum* (*LOL*1), *N. occultans*, *N. siegelii* and *N. uncinatum* (*LOL*1), all had closely related *LOL* clusters, which were inferred, therefore, to be derived from *E. bromicola* ancestors. Similarly, closely related *LOL* clusters in *N. coenophialum*, *N. uncinatum* (*LOL*2) and the *E. poae* × *E. elymi* hybrid from *Poa autumnalis* were inferred to be from *E. poae*. The *lolC* gene in *Neotyphodium* sp. FaTG-3 was related to the inferred *E. poaelolC* genes and to *lolC* from *N. aotearoae*, consistent with the *E. typhina* ancestor of FaTG-3. However, as was the case for *E. bromicola* and *E. poae*, no loline alkaloid producers [19] and no *LOL* genes were found in surveyed populations of *E. typhina*. The fourth ancestral contributor of a *LOL* cluster was *E. amarillans* (or a close relative), with which the *LOL* cluster in *E. canadensis* and the *LOL*2 cluster in *N. chisosum* grouped in clade IV.

Diversity of loline alkaloid profiles related to the presence or absence of certain late-pathway genes. Those that had the full suite of 11 *LOL* genes generally produced NFL and most or all of the other lolines including NANL, NML, loline, and AcAP. (The one exception is e4309, discussed later.) Furthermore, strains that lacked or had inactivating mutations in one late pathway *LOL* gene also had no functional copy of the downstream *LOL* genes. Hence, NANL producers *E. amarillans* E57, *E. canadensis* e4815, and *E. glyceriae* E277 lacked functional copies of *lolN*, *lolM* and *lolP*. Furthermore, *E. brachyelytri* E4804 and *E. canadensis* CWR34, which accumulated AcAP, lacked or had inactivating mutations in *lolO* as well as *lolN*, *lolM* and *lolP*. The only exception we observed to this pattern was *N. coenophialum* e4309, which had no obvious defect in any of the *LOL* genes and produced NANL, but no NFL. The basis for this chemotypic difference between e4309 and the NFL-producing *N. coenophialum* strains, such as e19 and e4163, is under investigation.

In every hybrid strain with two *LOL* clusters, one of the clusters was incomplete. In *N. uncinatum*, the *LOL*1 cluster from clade VI (*E. bromicola*) had all genes required for production of NFL, whereas the cluster from clade Ib lacked functional forms of three genes (*lolN*, *lolM*, and *lolP*) that were apparently required to take NANL to NFL. The clade Ib cluster had a remnant *lolP*, indicating that the capability to produce NFL may have been lost in the *E. poae* ancestor, rather than acquired in other clades. In support of this hypothesis, *N. coenophialum* e19 and e4163 both had *LOL* clusters from clade Ib that were complete, and both strains made NFL. The sequences of *LOL*2 genes in *N. uncinatum* were identical, or nearly so, to those of *N. coenophialum*. 

Similarly, *N. chisosum* had two *LOL* clusters. The cluster from clade VI was also complete in this strain, but the cluster from clade IV had an inactivating deletion in *lolP*, and lacked *lolN* and *lolM*. The deletion in *lolP* was identical to that in *E. amarillans* E57, consistent with the *LOL*1 copy originating from the clade IV ancestor. 

### 2.6. Alkaloid Cluster Compositions and Late-Pathway Gene Losses

Table 3 shows the clades of origin of the *EAS*, *IDT*, and *LOL* clusters in species and strains of hybrid epichloae. Table 4 lists the origins of individual genes in hybrids that have *EAS*, *IDT*, or *LOL* genes from more than one ancestor. In all of these cases, different ancestors contributed the genes for different alkaloids. Interestingly, genome sequencing consistently revealed that when two different ancestors contributed genes for the same class of alkaloids, those clusters always differed from each other in the presence or absence late-pathway genes. For example, FaTG-2 G2 strain NFe45079 had *IDT* genes from clades II and V. Although the IDT cluster originating from the *E. festucae* (clade II) ancestor had the two additional *IDT* genes for the final steps in lolitrem B biosynthesis (*ltmE* and *ltmJ*), there was no indication of such genes in clade V. The G3 genotype of FaTG-2 (strain NFe45115) lacked the clade II *IDT* genes, in keeping with the observation that it produces terpendoles but not lolitrem B [23]. This example illustrates that variation in homologous loci can lead to diversification of alkaloid profiles when clusters are subsequently lost. 

**Table 4:** Alkaloid biosynthesis gene origins in selected hybrid endophyte strains ^a^.

	*E. can.* e4815	*N. chi.* e3609	FaTG-2 G2	*N. coe.* e19	*N. coe.* e4163	*N. sie.* e915	*N. unc.* e167
Ergot alkaloids	ERV CC	nt	ERV	ERV CC	ERV	–	–
*dmaW*	III, IV	–	II,V	II,V	II,V	–	–
*easF*	III, IV	–	II,V	II,V	II,V	–	–
*easC*	III, IV	–	II,V	II,V	II,V	–	–
*easE*	III, IV	–	II,V	II,V	II	–	–
*easD*	IV	–	II,V	II,V	II,V	–	–
*easA*	IV	–	II,V	II,V	II,V	–	–
*easG*	IV	–	II,V	II,V	II,V	–	–
*cloA*	IV	–	II,V	II,V	II,V	–	–
*lpsB*	IV	–	+	IIΨ,V	II	–	–
*lpsA*	IV	–	+	II,V	II,V	–	–
*easH*	IV	–	II,V	II,V	II,V	–	–
Indole-diterpenes	–	–	TD, LTB	–	nt	nt	–
*idtG*	–	–	+	–	II	II	–
*idtC*	–	–	II,V	–	II	II	–
*idtM*	–	–	II,V	–	II	II	–
*idtB*	–	–	II,V	–	II	II	–
*idtS*	–	–	II,V	–	II	II	–
*idtP*	–	–	II,V	II	II	VI	–
*idtQ*	–	–	II,V	–	II	+	–
*idtF*	–	–	II,V	–	II	IIΨ	–
*idtK*	–	–	II,V	–	II	II	–
*idtE*	–	–	II	–	–	–	–
*idtJ*	–	–	II	–	–	–	–
Lolines	NANL	nt	–	NFL NAL NANL NML	NFL NAL NANL NML	NFL NAL NANL NML	NFL NAL NANL NML
*lolC*	IV	IV,VI	–	Ib	Ib	VI	Ib,VI
*lolF*	IV	IV,VI	–	Ib	Ib	VI	Ib,VI
*lolD*	IV	IV,VI	–	Ib	Ib	VI	Ib,VI
*lolT*	IV	IV,VI	–	Ib	Ib	+	Ib,VI
*lolU*	IV	IV,VI	–	Ib	Ib	+	Ib,VI
*lolO*	IV	IV,VI	–	Ib	Ib	+	Ib,VI
*lolE*	IV	IV,VI	–	Ib	Ib	VI	Ib,VI
*lolN*	–	VI	–	Ib	Ib	VI	VI
*lolM*	–	VI	–	Ib	Ib	+	VI
*lolP*	IV∆	IV∆,VI	–	Ib	Ib	VI	Ib∆,VI
*lolA*	IV	IV,VI	–	Ib	Ib	+	Ib,VI

^a^ Abbreviations: *E. can.* = Epichloë canadensis, FaTG-2 = Neotyphodium species FaTG-2, *N. chi* = Neotyphodium chisosum, *N. coe*. = Neotyphodium coenophialum, *N. sie*. = Neotyphodium siegelii, *N. unc*. = Neotyphodium uncinatum. - = not present, + = presence inferred from alkaloid profile or genotyping but clade relationship undetermined, nt = not tested, Ψ = pseudogene, ∆ = gene with large deletion rendering it nonfunctional. Other abbreviations as in Table 1 and Table 2.

The *EAS* and *LOL* clusters in hybrids showed a similar pattern. Both *N. chisosum* and *N. uncinatum* had one complete *LOL* gene cluster and a second cluster that lacked the three genes for conversion of NANL to NFL. Likewise, there were three examples of strains with two *EAS* clusters where only one had all genes known to be required for ergovaline production. In *E. canadensis* e4815 the clade IV *EAS* cluster was complete, but the clade III *EAS* cluster had only the four functional genes for biosynthesis of chanoclavine. Since the clade IV *EAS* cluster is absent in strain CWR34, plants with this strain accumulate chanoclavine but not ergovaline. Similarly, in *N. coenophialum* e4163, the *EAS*1 cluster lacked *easA* and *lpsB*, whereas in another *N. coenophialum* strain, e19, *EAS*1 was complete, and the *EAS*2 cluster had a defective *lpsB* gene. 

Based on the cluster maps in *E. festucae* and *N. coenophialum* e4163 (where the cluster sequences were best assembled), many of the genes for late pathway steps were located at the peripheries of the respective alkaloid gene clusters, and genes at such locations were often the ones that were absent from redundant clusters in hybrids. Specifically, *lpsB* and *easE* were at one end of the *EAS* cluster, *lolN* and *lolM* were at one end of the *LOL* cluster, *ltmE* and *ltmJ* were at one end of *IDT*, and *idtK* was at the other end of *IDT*. The exception was *lolP*, which was in the *LOL* core near early pathway genes. The observation that in all five examples of redundant alkaloid gene clusters (one for *IDT*,one for *LOL* and three for *EAS*) redundant genes for late pathway steps were absent raises the possibility that this pattern was determined by natural selection.

## 3. Discussion

The occurrence of alkaloid biosynthesis loci in the hybrid epichloae, as well as the relatively high levels of alkaloids that they produce in symbio [38,39], fits with theoretical predictions of the evolution of mutualism in heritable symbioses. The great majority of *Epichloë* and *Neotyphodium* species (epichloae) are capable of vertical, maternal-line transmission. Horizontal transmission in this group of fungi has so far been documented only for those that produce external stromata, manifesting abundant mitotic spores (conidia) or meiotic spores (ascospores) that can mediate infection of new plants or seeds [8,9,10]. Only one hybrid species, *Epichloë liyangensis*, has ever been observed to produce stromata [40], so it appears that the hybrids are generally dependent on vertical transmission. Such a situation is expected to select for mutualism because the fitness of a vertically transmitted symbiont links positively to host fitness [41,42]. A common benefit conferred by epichloae to their hosts is the production of protective alkaloids that can deter, impair or even kill invertebrate and vertebrate herbivores [1,3,4,22,43,44,45,46,47,48,49,50,51,52,53]. Whenever a hybrid forms, its fitness may depend on the alkaloid biosynthesis capabilities that it has obtained from its ancestors. Such selection on hybrids may explain a curious finding of this study; that is, surveys of *E. bromicola*, *E. poae*, and *E. typhina* have failed to turn up isolates with *LOL* genes, and in sequenced genomes of those species there was no indication of remnant *LOL* genes of any sort. Yet, these three sexual species or close relatives were the apparent donors of *LOL* clusters to five of the six loline-producing hybrid species in this study. Thus, it appears that selection has served as a highly effective filter for hybrid epichloae with alkaloid production capabilities.

The contrast of apparently low frequencies of *LOL* genes in *E. bromicola*, *E. poae*, and *E. typhina*, versus the existence of several hybrids that possess *LOL* genes from one or more of these species, begs the question whether hybrid, asexual epichloae have more tendency to produce protective alkaloids than do sexual non-hybrids. The vast majority of hybrid epichloae surveyed to date have alkaloid biosynthesis genes. For instance, of 32 isolates from 24 hybrid species surveyed earlier, 26 isolates (81%) from 20 species have *EAS*, *IDT* or *LOL* genes [24]. In contrast, 53 isolates from ten sexual species have been surveyed, of which 23 isolates (43%) from eight species test positive for the determinant gene in each cluster. This is a slight downward revision from the number of positives reported previously in sexual isolates [24], but follow-up investigations indicated that at least four cases of positive tests were due to paralogs that are probably not involved in those alkaloid pathways (for example, the *dmaW* paralog in *E. baconii* E1031). Although these surveys cannot be considered unbiased or random, they are suggestive that selection for alkaloid production might be stronger on epichloae that are strictly vertically transmitted, and such a hypothesis is worth further investigation. 

It is intriguing that whenever hybrids had two homologous gene clusters for an alkaloid, one of the clusters lacked some late pathway genes. This was true of all such hybrids for which, by genome sequencing, we could conduct a comprehensive analysis of all of the alkaloid gene clusters. We can speculate on why this pattern is so common. One possibility is that it arose simply by chance and might simply reflect the frequent occurrence of various forms of those clusters in the ancestors of the hybrids. In fact, the alkaloid gene clusters of epichloae seem especially suited to gene losses because of their extensive tracts of interspersed repeats [6]. In the case of *N. coenophialum*, it appears that two ancestors contributed complete *EAS* clusters, and as lineages then diverged, some lost certain genes, others lost different genes, and in some strains (e.g., e4309) the *EAS* genes were altogether lost. These gene losses may just reflect an absence of selection to maintain them, and in the cases of strains e19 and e4163, the remaining complete cluster complements the defects. But, if these situations are simply random, why was there a tendency for late-pathway genes to be lost? 

An intriguing alternative reason behind the selective loss of redundant late-pathway genes could be that the resulting alkaloid profiles provide added benefit to the host grass and, by extension, to the symbiont (again, because of reliance on vertical transmission). Panaccione [54] hypothesized that alkaloid pathways may be less than maximally efficient to allow for accumulation of a variety of pathway intermediates. These intermediates may differ in biological activities, so that combinations of bioactive compounds may confer additive or synergistic activities against herbivores and parasites. Reduplication of an alkaloid gene cluster by hybridization may lead to increased efficiency, thereby reducing levels of intermediates and spur products relative to the pathway end product. In contrast, eliminating late pathway genes from just one of two homologous clusters in a genome might cause a reduced efficiency of the late pathway steps relative to early pathway steps, resulting in a more complex mixture of alkaloids. In support of this hypothesis, most plants with endophytes predicted to produce ergovaline have intermediates as well, particularly chanoclavine [54]. Likewise, NFL producers have several loline alkaloid intermediates, often with substantial levels of NANL [55,56,57]. And, the indole-diterpene pathway is no exception, with numerous intermediates and spur products evident in plants with endophytes that make lolitrem B [22]. Therefore, future surveys should also be designed to address the question whether certain alkaloid profiles, including abundant expression of intermediates and spur products, might be selectively favored over simpler profiles dominated by pathway end products.

## 4. Materials and Methods

### 4.1. Genome Sequencing

Fungal DNA was isolated and sequenced by pyrosequencing on a Roche/454 GS FLX+ platform as described previously [6]. The genome of *N. coenophialum* was sequenced by a combination of pyrosequencing and Sanger sequencing of fosmid-cloned ends, as done previously for *E. festucae* Fl1 [6]. The extended-read protocol was used for isolates Lp1, E1031, e4815, e3609, e4096, e4163, E3601, and E4668, giving average high-quality read lengths ranging from 646 to 779 nt.

### 4.2. Amplicon Sequencing of Alkaloid Biosynthesis Genes

Those strains for which comprehensive genome sequence information was lacking were first genotyped by PCR with primers designed against genes for key alkaloid biosynthesis pathway steps [23,26]. Then, alkaloid gene fragments were PCR-amplified and sequenced from isolated DNA of cultures or symbiotic plant tissue. Genomic DNA was isolated from lyophilized mycelium of FaTG-2 isolates NFe45079 and NFe45115, and FaTG-3 isolate NFe1100 using the ZR fungal/bacterial DNA Miniprep kit (Zymo Research, Irvine, CA, USA), and quantified by fluorometry with Hoechst dye staining using a DyNA Quant 200 fluorometer (Hoefer, Inc., Holliston, MA, USA). In the cases of *Neotyphodium siegelii* e915 and *Epichloë canadensis* CWR5 and CWR34, DNA was isolated from endophyte-infected tillers, and DNA was also isolated from seeds of annual ryegrasses (*Lolium* spp.) symbiotic with *Neotyphodium occultans*. Total DNA from endophyte-infected plant materialwas isolated using a MagAttract 96 DNA plant core kit (Qiagen, Valencia, CA, USA). DNA extracted in this manner was not quantified prior to amplification. 

Alkaloid biosynthesis gene fragments were amplified from total DNA extracted from fungal mycelia (5 ng), endophyte-infected tillers (3 µL) or seeds (6 µL) using the primers designed against conserved regions of the genes [23,26]. PCR mixtures included 1× Green GoTaq reaction buffer (Promega, Madison, WI, USA), 200 µM each deoxynucleoside triphosphate (dNTP), 200 nM each primer, and 1 U GoTaq DNA polymerase (Promega). PCR amplifications were conducted under the following conditions: 94 °C for 2 min; 30 cycles of 94 °C for 15 s, 56 °C for 30 s, and 72 °C for 2 min; and then 1 cycle of 72 °C for 7 min. After amplification, an aliquot (5–10 µL) of the mixture was visualized by agarose gel electrophoresis to confirm the final products were of expected size. The remaining reaction volume was purified using a QIAquick PCR Purification Kit (Qiagen, Valencia, CA, USA). Purified PCR amplicons were sequenced using BigDye chemistry (v3.1; Applied Biosystems, Foster City, CA, USA) and analyzed with an Applied Biosystems 3730 DNA Analyzer. Sequences were viewed, edited, and managed using Sequencher v5.0 (Gene Codes, Ann Arbor, MI, USA). 

### 4.3. Alkaloid Analyses

Methods of alkaloid analysis were as described previously for ergot alkaloids [20], lolines [58], and indole-diterpenes [21].

## 5. Conclusions

Epichloae (*Epichloë* and *Neotyphodium* species) are systemic symbionts of cool-season grasses (Poaceae, subfamily Poöideae), and most are capable of disseminating via seeds to successive generations of host plants (vertical transmission). Whereas sexual strains can also spread contagiously via spores (horizontal transmission), asexual strains are largely or exclusively seed-borne. Vertically transmissible epichloae can be highly beneficial to their hosts, and many of these mutualistic symbionts are interspecific hybrids with two or three ancestors among the sexual species. Host benefits relate in large part to the ability of epichloae to produce alkaloids that help deter herbivory by vertebrates and invertebrates. Genome sequence comparisons between epichloae species and strains revealed alkaloid clusters of genes for biosynthesis of ergot alkaloids (*EAS*), indole-diterpenes (*IDT*), and lolines (*LOL*). Alkaloid production is more commonly reported in asexual than in sexual epichloae, so we undertook to determine from where 12 different hybrid species had derived their alkaloid biosynthesis genes. As expected, phylogenetic relationships of alkaloid genes in hybrids matched the phylogeny of a housekeeping gene. Surprisingly, six hybrid species had *LOL* from either or both of the sexual species, *Epichloë bromicola* or *E. poae*, even though loline genes were not found in surveys of these sexual ancestors. This result suggests that hybrids possessing these alkaloid genes may have had a selective advantage over strains that did not. Another surprising observation was that in all five hybrids with duplicate copies of *EAS*, *IDT*, or *LOL* gene clusters, some late pathway genes were absent from one of the redundant clusters. We speculate that this pattern reflects selection favoring alkaloid profiles with substantial levels of biosynthetic intermediates in addition to the pathway end-products.

## Supplementary Files

Supplementary File 1 Supplementary Information (PDF, 147 KB)
