# Supplementary material for: Currencies of Mutualisms: Sources of Alkaloid Genes in Vertically Transmitted Epichloae
Source: Toxins (Basel). 2013 Jun 6;5(6):1064–88. doi: 10.3390/toxins5061064 (PMC3717770; doi:10.3390/toxins5061064)
Supplement: Supplementary File 1 — Supplementary Information (PDF, 147 KB) [file toxins-05-01064-s001.pdf]

Supplementary Information

Table S1. *tubB* genes featured in Figure 3.

Species	Isolate [a]	Host	*tub2*
Epichloë amarillans	E273	*Agrostis hyemalis*	AF457466
E. amarillans	E57 = ATCC 200744	*Ag. hyemalis*	KF042047
E. amarillans	E4668	*Ag. hyemalis*	KF042042
E. amarillans	E1087	*Elymus virginicus*	AF457478
E. amarillans	ATCC 200743	*Sphenopholis obtusata*	L06958
E. amarillans	ATCC 201670	*Sph. obtusata*	AF062426
Epichloë baconii	ATCC 76552	*Agrostis stolonifera*	L06961
E. baconii	ATCC 90167	*Agrostis tenuis*	L78279
E. baconii	ATCC 200746	*Ag. tenuis*	AF250733
E. baconii	E1031 = ATCC 200745	*Calamagrostis villosa*	KF042062
Epichloë brachyelytri	ATCC 200752	*Brachyelytrum erectum*	L78271
E. brachyelytri	ATCC 200753	*Be. erectum*	AF250734
E. brachyelytri	ATCC 200754	*Be. erectum*	AF250735
E. brachyelytri	ATCC 201560	*Be. erectum*	AF250736
E. brachyelytri	ATCC 201561	*Be. erectum*	AF062427
E. brachyelytri	E4804	*Be. erectum*	KF042060
Epichloë bromicola	AL0814	*Agropyron repens*	GU325782
E. bromicola	AL9121/1	*Bromus erectus*	AY033378
E. bromicola	AL9607	*Bro. erectus*	AY033366
E. bromicola	AL9608	*Bro. erectus*	AY033381
E. bromicola	AL9630	*Bro. erectus*	AY033382
E. bromicola	AL9631	*Bro. erectus*	AY033383
E. bromicola	AL9632	*Bro. erectus*	AY033384
E. bromicola	AL9633	*Bro. erectus*	AY033385
E. bromicola	ATCC 200749	*Bro. erectus*	L78289
E. bromicola	E502 = ATCC 200750	*Bro. erectus*	KF042058
E. bromicola	DEB 9831/4	*Bro. erectus*	AY033365
E. bromicola	ATCC 201558	*Bro. ramosus*	KC936102
Epichloë canadensis	CWR 5	*Elymus canadensis*	JN886778<br>JN886777
Epichloë clarkii	ATCC 200741	*Holcus lanatus*	AF250738
E. clarkii	ATCC 200742	*H. lanatus*	L78281
Epichloë elymi	E1081	*Bromus kalmii*	AF457477
E. elymi	E56 = ATCC 201551	*Elymus canadensis*	KF042052
E. elymi	ATCC 200850	*Elymus virginicus*	L78273
E. elymi	ATCC 201553	*El. virginicus*	AF062428
E. elymi	ATCC 201554	*El. virginicus*	AF250742
E. elymi	ATCC 201556	*Elymus hystrix*	AF250744
E. elymi	ATCC 201557	*El. hystrix*	AF250745
E. elymi	E4132	*El. hystrix*	AF457468
Epichloë festucae	E28	*Festuca trachyphylla*	L06956
E. festucae	ATCC 201550	*Festuca rubra* subsp. *commutata*	L06957
E. festucae	ATCC 90660	*F. rubra* subsp. *commutata*	AF250746
E. festucae	ATCC 90661	*F. rubra subsp. rubra*	L06955
E. festucae	E1157	*Koeleria pyramidata*	AF250747

Table S1. *Cont.*

**Species**	**Isolate** [a]	**Host**	***tub2***
E. festucae	E434	*Lolium giganteum*	L78286
E. festucae	E2368	*F. rubra* subsp. *rubra*	KF042044
E. festucae	Fl1	*Festuca trachyphylla*	KF042045
Epichloë glyceriae	E277 = ATCC 200747	*Glyceria striata*	KF042046
E. glyceriae	E2772 = ATCC 200755	*G. striata*	L78276
Epichloë poae	e187	*Poa secunda* subsp. *junicifolia*	JQ756453
E. poae	ATCC 201667	*Poa nemoralis*	AF062429
E. poae	ATCC 201668	*Poa nemoralis*	AF250756
E. poae	E5100	*Poa nemoralis*	KC936115
E. poae	E5102	*Poa nemoralis*	KC936114
E. poae	E5361	*Poa nemoralis*	KC936113
E. poae	E5819	*Poa nemoralis*	KF042043
E. poae	ATCC 201669	*Poa pratensis*	L78284
E. poae	E1154 = CBS 102655	*Poa pratensis*	AF250757
Epichloë sylvatica	ATCC 200748	*Brachypodium sylvaticum*	L78278
E. sylvatica	ATCC 200751	*Bp. sylvaticum*	L78291
E. sylvatica	Brhs6402	*Bp. sylvaticum*	KC936106
E. sylvatica	Brhs6410	*Bp. sylvaticum*	KC936107
E. sylvatica	Brhs6710	*Bp. sylvaticum*	KC936108
E. sylvatica	Brhs6914	*Bp. sylvaticum*	KC936109
Epichloë typhina	E7358	*Achnatherum pekingensis*	KC936101
E. typhina	ATCC 200738	*Anthoxanthum odoratum*	L78288
E. typhina	TC1	*Brachypodium phoenicoides*	AM490796
E. typhina	ATCC 200739	*Brachypodium pinnatum*	L78292
E. typhina	E5232	*Bp. pinnatum*	KC936111
E. typhina	E5240	*Bp. pinnatum*	KC936105
E. typhina	E5303	*Bp. pinnatum*	KC936112
E. typhina	E5319	*Bp. pinnatum*	KC936110
E. typhina	ATCC 200740	*Dactylis glomerata*	L78274
E. typhina	E5261	*D. glomerata*	KC936130
E. typhina	E5268	*D. glomerata*	KC936128
E. typhina	E5272	*D. glomerata*	KC936121
E. typhina	E5333	*D. glomerata*	KC936132
E. typhina	E5347	*D. glomerata*	KC936124
E. typhina	E5350	*D. glomerata*	KC936118
E. typhina	E5070	*D. glomerata*	KC936122
E. typhina	E5071	*D. glomerata*	KC936139
E. typhina	E5075	*D. glomerata*	KC936119
E. typhina	E5076	*D. glomerata*	KC936129
E. typhina	E5077	*D. glomerata*	KC936140
E. typhina	E5078	*D. glomerata*	KC936136
E. typhina	E5079	*D. glomerata*	KC936141
E. typhina	E5084	*D. glomerata*	KC936134
E. typhina	E5088	*D. glomerata*	KC936116
E. typhina	E5089	*D. glomerata*	KC936137

Table S1. *Cont.*

Species	Isolate [a]	Host	*tub2*
E. typhina	E5090	*D. glomerata*	KC936142
E. typhina	E5092	*D. glomerata*	KC936120
E. typhina	E5094	*D. glomerata*	KC936138
E. typhina	E5096	*D. glomerata*	KC936133
E. typhina	E5142	*D. glomerata*	KC936123
E. typhina	E5143	*D. glomerata*	KC936131
E. typhina	E5146	*D. glomerata*	KC936127
E. typhina	E5159	*D. glomerata*	KC936135
E. typhina	E8 = ATCC 200736	*Lolium perenne* subsp. *perenne*	X52616
E. typhina	E432	*L. perenne* subsp. *perenne*	AF250752
E. typhina	ATCC 200851	*Phleum pratense*	L78280
E. typhina	CBS 102658	*Phleum pratense*	AF250753
E. typhina	E348 = CBS 102648	*Phleum pratense*	L78277
E. typhina	E5001	*Poa trivialis*	KC936103
E. typhina	E5002	*Poa trivialis*	KC936104
E. typhina	AL0525/2	*Puccinellia distans*	EU375739
E. typhina	AL9725	*Puc. distans*	EU375740
E. typhina (syn. *E. poae*) var. *aonikenk*	Bs420	*Bromus setifolius*	AY707694
E. yangzii	Rnj4201	*Roegneria kamoji*	DQ134039
Epichloë sp.	E3601 = AL9924	*Holcus mollis*	KF042061
FaTG-2 (G2)	NFe45079	*Lolium arundinaceum*	X028253<br>JX028254
FaTG-2 (G3)	NFe45115	*L. arundinaceum*	JX028255<br>JX028256
FaTG-3	e4074	*Lolium* sp.	L20308<br>L06952
FaTG-3	NFe1100	*Lolium* sp.	KF030703<br>KF030704
FaTG-4	e4305	*Festuca arundinacea* var. *letourneuxiana*	KC936117<br>KC936143
N. aotearoae	MYA-1193	*Echinopogon ovatus*	AF323371
N. aotearoae	e899 = MYA-1229	*Ec. ovatus*	KF042049
N. chisosum	e3609 = ATCC 64037	*Achnatherum eminens*	AF457470<br>AF457471<br>AF457472
N. coenophialum	e19 = ATCC 90664	*Lolium arundinaceum*	KF036272<br>KF036273<br>KF036274
N. coenophialum	e4163	*L. arundinaceum*	KF036275<br>KF036276<br>KF036277
N. coenophialum	e4309	*L. arundinaceum*	KF036278<br>KF036279<br>KF036280

Table S1. *Cont.*

Species	Isolate [a]	Host	*tub2*
N. funkii	e4096	*Achnatherum robustum*	AF457489<br>AF457490
N. gansuense	e7080	*Achnatherum inebrians*	KF042053
N. gansuense	e7082	*A. inebrians*	EF422757
N. gansuense	e7083	*A. inebrians*	EF422759
N. gansuense	e7084	*A. inebrians*	EF422758
N. gansuense	e7085	*A. inebrians*	EF422760
N. gansuense	e7086	*A. inebrians*	EF422761
N. gansuense	e7087	*A. inebrians*	EF422762
N. gansuense	e7088	*A. inebrians*	EF422763
N. gansuense	e7090	*A. inebrians*	EF422764
N. gansuense	e7092	*A. inebrians*	EF422765
N. gansuense var. *inebrians*	e817	*A. inebrians*	AF457495
N. gansuense var. *inebrians*	e818 = ATCC MYA-1228	*A. inebrians*	KF042059
N. lolii	e135 = Lp19	*Lolium perenne*	KC936144
N. lolii x *E. typhina* (LpTG-2)	e144 = Lp1	*L. perenne*	L20304<br>L78286
N. occultans	Lm2	*Lolium multiflorum*	AF176268<br>AF176274
N. occultans	Lrr1	*L. rigidum* var. *rigidum*	AF176271<br>AF176275
N. occultans	Lrr2	*L. rigidum* var. *rigidum*	AF176270<br>AF176276
N. occultans	e992	*L. rigidum* var. *rigidum*	AF176271<br>AF176275
N. occultans	Lro1	*L. rigidum* var. *rottboellioides*	AF176269<br>AF176272
N. sibiricum	MTI-H50	*Achnatherum sibiricum*	GQ421698
N. sibiricum	MTI-X47	*A. sibiricum*	GQ421706
N. sibiricum	MTI-X85	*A. sibiricum*	GQ421707
N. sibiricum	A-LB	*A. sibiricum*	DQ675589
N. siegelii	E915 = ATCC 74483	*Lolium pratense*	AF308138<br>AF308139
N. stromatolongum	Cnj6617	*Calamagrostis epigeios*	EU526824
N. stromatolongum	Cnj6620	*C. epigeios*	EU526825
N. uncinatum	E167 = CBS 102646	*L. pratense*	KF042048
PauTG-1	e55	*Poa autumnalis*	AF457473<br>AF457474

[a] Strain designations beginning ATCC or MYA are from the American Type Culture Collection, strain designations beginning CBS are from the Centraalbureau voor Schimmelcultures, and other designations are from the laboratories where the strains were isolated.

Table S2. GenBank accession numbers for alkaloid biosynthesis genes.[a]

Organism	Strain	*EAS*	*IDT/LTM*	*LOL*
Epichloë amarillans	E57 = ATCC 200744	-	-	JF830812, JF830813
E. amarillans	E4668	KC989564, KC989563	-	KC990436
Epichloë baconii	E1031 = ATCC 200745	KC989571	-	KC990439, KC990440
Epichloë brachyelytri	E4804	JN378894, JN378895, JN378896	-	JF800660, JF800661, JF800659
Epichloë bromicola	E502 = ATCC 200750	-	-	-
Epichloë canadensis	e4815	KC989568, KC989567, KC989566, KC989565, KC989612	-	KC990447, KC990443, KC990444, KC990445, KC990446
E. canadensis [b]	CWR 5	KC989604, KC989603, KC989602	-	KC969638, KC969639, KC969640
E. canadensis [b]	CWR 34	KC989606, KC989605	-	KC969635, KC969636, KC969637
Epichloë elymi	E56 = ATCC 201551	JX439640, JX439641, JX439642	-	-
Epichloë festucae	E2368	JN167225, JN167226, JN167227	JX402753	JF830815, JF830814, JF830816
Epichloë festucae	Fl1	JN177500, JN177501, JN177502	JN613318, JN613319, JN613320	-
Epichloë glyceriae	E277 = ATCC 200747	JN177503, JN177504, JN177505, JN177506	-	JF800664, JF800665, JF800663
***Epichloë* sp.**	E3601 = AL9924	KC989574, KC989573, KC989572,	-	-
Epichloë poae	E4646	-	-	-
E. poae	E5819	JN182230, JN182231, JN182232	-	-
Epichloë typhina	E8 = ATCC 200736	-	-	-

Table S2. *Cont.*

Organism	Strain	*EAS*	*IDT/LTM*	*LOL*
***Neotyphodium* sp. FaTG-2, G2 genotype [b]**	NFe45079	KC989601, KC989600, KC989599, KC989598, KC989597, KC989596, KC989595, KC989594, KC989593, KC989592	KC970517, KC970518, KC970523, KC970524, KC970528, KC970529, KC970541, KC970544, KC970545, KC970552, KC970553, KC970560, KC970561, KC970562, KC970563, KC970570, KC970571,	-
***Neotyphodium* sp. FaTG-2, G3 genotype [b]**	NFe45115	KC989591, KC989590, KC989589, KC989588, KC989587, KC989586, KC989585	KC970525, KC970530, KC970536, KC970540, KC970543, KC970551, KC970559, KC970564, KC970567	-
***Neotyphodium* sp. FaTG-3 [b]**	NFe1100	-	KC970522, KC970527, KC970535, KC970539, KC970546, KC970550, KC970558, KC970565, KC970568	KC969615, KC969616, KC969617, KC969618, KC969619
***Neotyphodium* sp. FaTG-3**	e4074	-	-	KF027206
***Neotyphodium* sp. FaTG-4**	e4305	KC989581, KC989580, KC989579, KC989578, KC989577, KC989576, KC989575	KC970537, KC970573, KC970582, KC970579	-

Table S2. *Cont.*

Organism	Strain	*EAS*	*IDT/LTM*	*LOL*
Neotyphodium aotearoae	e899 = MYA-1229	-	KC970519, KC970555, KC970574, KC970575, KC970576, KC970577	KC990449, KC990451, KC990450, KC990448,
Neotyphodium chisosum	e3609 = ATCC 64037	-	-	KC990454, KC990455, KC990452, KC990453, KC990456,
Neotyphodium coenophialum	e19 = ATCC 90664	KC989611, KC989610, KC989609, KC989608, KC989607	KC970554	EF012268 KF027203, KF027204, KF027205
N. coenophialum	e4163	KC989570, KC989569	KC970578	KC990458, KC990457, KC990459
N. coenophialum	e4309	-	KC970572, KC970581, KC970584	KC990460, KC990462, KC990461
Neotyphodium funkii	e4096	KF042039, KF042040, KF042041	KC920437, KC920438	-
Neotyphodium gansuense	e7080	-	JN587271	JF800666
N. gansuense* var. *inebrians	e818 = MYA-1228	JX072969, JX273434	JX072969	-
Neotyphodium occultans	e999	nd	nd	KF039908
N. occultans [b]	Non-culturable From *Lolium temulentum* GRIN PI 415813	-	KC970520, KC970531, KC970532, KC970533, KC970547, KC970548, KC970557, KC970566	KC969629, KC969630, KC969631, KC969632, KC969633

Table S2. *Cont.*

Organism	Strain	*EAS*	*IDT/LTM*	*LOL*
Neotyphodium siegelii [b]	e915 = ATCC 74483	-	KC970521, KC970526, KC970534, KC970538, KC970542, KC970569 KC970549, KC970556	KC969627, KC969624, KC969625, KC969626, KC969628
Neotyphodium uncinatum	e167 = CBS 102646	-	-	AY723749, AY723750, AY724686, JX430081, JX430082
Neotyphodium lolii	e135 = Lp19	EF125025	AY742903, DQ443465	-
N. lolii x E. typhina	e144 = Lp1	KC989584, KC989583, KC989613, KC989582	KC970580, KC970583	-
***Neotyphodium* sp. PauTG-1**	e55	-	-	EF012269, EF015399

[a] nd = not determined; - = absent; [b] Data generated through amplicon sequencing.

© 2013 by the authors; licensee MDPI, Basel, Switzerland. This article is an open access article distributed under the terms and conditions of the Creative Commons Attribution license (http://creativecommons.org/licenses/by/3.0/).
